# Exercise monitoring from a Network Physiology approach

**DOI:** 10.3389/fnetp.2026.1745246

**Published:** 2026-10-29

**Authors:** Lluc Montull, Sergi Garcia-Retortillo, Óscar Abenza, Yaopeng J. X. Ma, Robert Hristovski, Plamen Ch. Ivanov, Natàlia Balagué

**Affiliations:** 1 National Institute of Physical Education of Catalonia (INEFC Pirineus), University of Lleida, La Seu d’Urgell, Spain; 2 College of Nursing, University of Central Florida, Orlando, FL, United States; 3 Faculty of Medicine and Health Sciences, University of Barcelona, Barcelona, Spain; 4 Keck Laboratory for Network Physiology, Department of Physics, Boston University, Boston, MA, United States; 5 Faculty of Physical Education, Sport and Health, University Ss. Cyril and Methodius, Skopje, North Macedonia; 6 Institute of Solid State Physics, Bulgarian Academy of Sciences, Sofia, Bulgaria; 7 Department of Neurosurgery, Boston University Chobanian and Avedisian School of Medicine, Boston, MA, United States; 8 National Institute of Physical Education of Catalonia (INEFC Barcelona), University of Barcelona, Barcelona, Spain

**Keywords:** complex adaptive systems, exercise, inter-muscular networks, inter-organs networks, intra-individual variability, network physiology, nonlinear dynamics, sports science

## Abstract

Advancements in big data, artificial intelligence and related technology are shaping the future of exercise monitoring, yet current monitoring strategies may not fully capture the physiological complexity of athletes’ health and performance. The multidisciplinary field of Network Physiology provides a conceptual and analytical framework to investigate the dynamic interactions, coordination and network integration among physiological systems in athletes during exercise, and thus, opens new directions in Exercise Physiology. This article aims to summarize the current advances, vision and perspectives within this framework, focusing on how investigations of the inter-organ dynamic networks (cardio-respiratory, cardio-muscular, cortico-cardiac, cortico-muscular, inter-muscular, etc.) can lead to valuable insights into athletes’ performance, exercise monitoring, the underlying regulatory mechanisms of systems adaptation and coordination in response to exercise-induced stress, and to identify a novel class of network-based biomarkers related to training, levels of fitness, fatigue, and recovery.

## 1 Introduction

Recent advancements in exercise monitoring have significantly improved the ability to analyze and enhance athletic performance while prioritizing the health and wellbeing of athletes at different levels of fitness and training (Foster et al., 2017). However, current conceptual, methodological, and technological limitations reduce the efficacy and sensitivity of exercise monitoring.

Conceptually, athletes have been traditionally approached as machine-like devices composed of weakly interacting component modules with often linear and predictable responses. In recent decades, a growing body of research has reconceptualized the organism as a complex adaptive system exhibiting previously unrecognized properties such as self-organization, synergies^1^, context-dependency or nonlinearity (Hristovski, 1996; Balagué et al., 2020; Pol et al., 2020).

Methodologically, exercise monitoring has often relied on mechanistic and reductionist models, oversimplifying the complex and dynamic nature of athletes’ physiology (Balagué et al., 2020; Pol et al., 2020). Physiological, psychological, biomechanical, and performance variables are commonly monitored and interpreted separately in most current exercise monitoring systems (Halson, 2014; Bourdon et al., 2017). In recent years, there is a growing trend in developing integrative frameworks and tools using big data sources (e.g., omics), relational measures (e.g., correlation between HR and VO2 kinetics during recovery), and artificial intelligence to obtain pertinent and actionable information (Vianna et al., 2014; Heidari et al., 2019; Fiscutean, 2021; Sellami et al., 2022; Taber et al., 2024). In such a general scenario, several limitations should be highlighted.

Many measures still lack to assess the time-varying (dynamic) structure of physiological systems and the time-varying interactions among multiple physiological systems simultaneously. Predictive models that lack this information should be interpreted with caution (Hu et al., 2016; Ching et al., 2018; Dergaa et al., 2023). In complex adaptive systems, predictive models may have limited accuracy because these systems exhibit stochastic and indeterministic nonlinear dynamics (Kelso, 1995; Ivanov et al., 1998; Schulte-Frohlinde et al., 2001; Ashkenazy et al., 2002; Fuchs and Jirsa, 2008; Glass, 2009). Therefore, an important limitation of such predictive approaches lies in the inability of the input variables to represent the time-varying temporal organization in the dynamics of physiological systems.

In this context, available monitoring devices are often programmed to calculate maximal discrete values and averaged distributions, which might ignore the intra-individual systems dynamics and not take into account the non-stationarity of physiological signals (Balagué et al., 2020; Pol et al., 2020). In recent decades, there has been a growing body of work with a focus on quantifying and assessing the importance of temporal variability in physiological signals, such as heart rate variability (HRV), to understand the underlying control mechanisms (Ivanov et al., 1996; Ivanov et al., 1998; Gronwald et al., 2019). The cardiac system provides a paradigmatic example of how physiological variability came to be understood as complex and multiscale. Studies revealed long-range correlations and crossover phenomena in heartbeat dynamics (Peng et al., 1995), state-dependent scaling and multifractal organization (Ivanov et al., 1999a; Ivanov et al., 1999b; Ivanov et al., 2001), and multiscale cardiac control (Ivanov et al., 2004; Ivanov, 2007). Modeling studies further showed how stochastic feedback and transient correlations can generate complex physiological variability across a range of time scales (Ivanov et al., 1998; Kantelhardt et al., 2003). Importantly for exercise physiology, heartbeat temporal correlations reorganize with transitions from rest to exercise (Karasik et al., 2002).

Previous research has suggested that temporal variability measures of physiological variables might be more sensitive than threshold, mean, or maximal values in detecting relevant nonlinear events related to exercise, such as acute fatigue (Yassierli and Nussbaum, 2008; Gronwald et al., 2019) or injuries (Neumann et al., 2024). That is, they allow capturing the subtle transitions between a variety of physiological states, which cannot be simplified into dichotomous categories like trained-untrained, healthy and unhealthy, injured and non-injured, or fatigued and non-fatigued (Sturmberg et al., 2019).

The critical fluctuations of physiological or kinematic variables have been used as early warning signals to predict transitions towards unhealthy states (Chevance et al., 2021), injuries (Neumann et al., 2024), overtraining (Tian et al., 2013) or task disengagement due to accumulated effort (Hristovski and Balagué, 2010; Vázquez et al., 2016; Vázquez et al., 2021). That is, it seems that temporal variability measures may offer probabilistic insights into an athlete’s state, allowing for some degree of forecasting of future behaviors based on the stability or instability of current states (Scheffer et al., 2009).

Technologically, still some marker recordings such as blood, sweat, or urine remain largely limited to static evaluations (i.e., one data point) or low sampling rates of data collection. Advancements in wearable technology are expanding possibilities for acquiring recordings of physiological variables at high sampling rates**.** These include biopotential measures like electromyography (EMG), electrocardiography (ECG) or electroencephalography (EEG), as well as other measures like near-infrared spectroscopy (NIRS and fNIRS), oximeters, transthoracic impedance, blood pressure sensors, galvanic skin response, sweat sensors, glucose monitor, body temperature, respiratory sensors or photoplethysmography (Bowler et al., 2022; Rabost-Garcia et al., 2023; Seçkin et al., 2023). These technologies, allowing for continuous time-series recordings, permit to assess dynamic properties of physiological processes, for instance, how distinct variables coordinate over time (Bashan et al., 2012; Kerkman et al., 2018; Garcia-Retortillo et al., 2023) or show critical fluctuations (Casties et al., 2006; Bartlett et al., 2007; Bradley and Kantz, 2015). However, low sampling rates and challenges in synchronizing certain physiological measures constrain this type of assessment.

Pioneering studies on physiological interactions showed that the coupling between organ systems is dynamic, state-dependent, and may exhibit distinct coexisting forms and transitions (Chen et al., 2006; Bartsch et al., 2012; Bartsch et al., 2014; Bartsch and Ivanov, 2014). Such findings provided an important basis for moving beyond pairwise coupling and towards the study of how physiological systems with distinct control mechanisms integrate as dynamic adaptive networks to coordinate functions. This lead to the emergence of Network Physiology—a field providing a conceptual and analytical framework—to investigate how physiological states and functions at the organism level emerge from systems interactions and their collective organization as a network (Bashan et al., 2012; Ivanov and Bartsch, 2014; Bartsch et al., 2015; Liu et al., 2015b; Ivanov et al., 2017). Consequently, Network Physiology of Exercise developed within this broader framework to investigate the dynamic mechanisms of coordination and network integration among physiological systems during exercise, and the evolution of physiological networks with training, levels of fitness, fatigue, and recovery (Balagué et al., 2020; Balagué et al., 2022b; Balagué et al., 2022a). Further, Network Physiology seeks to develop dynamic network-based markers to gather integrative insights into horizontal interactions (among organs) and vertical interactions (among scales and levels of organization from genomic, metabolic, and cellular interactions to tissues and organs) to assess the organism’s multilevel functional state (Ivanov, 2021). Research in the field has utilized biopotential signals from organs with high-frequency sampling rates, which has revealed markedly different patterns of dynamic coupling among the cardiac, respiratory, cortical, and muscular systems, such as differences in coupling strength and degree of synchronization, temporal stability, directionality, and causality (Bartsch et al., 2015; Faes et al., 2015b; Faes et al., 2015a; Lin et al., 2016; Rizzo et al., 2020; Ivanov et al., 2021a).

Network-based markers can improve current monitoring systems with highly sensitive assessments of exercise-related phenomena. For example, Garcia-Retortillo et al. (2017) showed in healthy active adults that cardio-respiratory coordination was more sensitive to the effects of fatigue in repeated maximal exercises than conventional physiological measures like VO_2_ or ventilation threshold. Further, Abenza et al. (2025b) showed that cardio-respiratory coordination could differ between healthy adults with higher and lower exercise recovery capacity, while ventilation and VO_2_ values at the end of the exercise recovery did not. Similarly, Papadakis et al. (2022) suggested that cardio-respiratory coordination was more closely related to the performance of rowers compared to VO_2max._

Against this background, this article aims to summarize the current advances, vision and perspectives within the Network Physiology framework, focusing on how inter-organ dynamic networks (cardio-respiratory, inter-muscular, cardio-muscular, cortico-muscular, cortico-cardiac) may offer valuable insights to exercise monitoring.

## 2 Network-based markers

A network is any discrete system which may be represented as a graph G = (N, L, W), where: N is a set of nodes (vertices); L is a set of links or edges connecting the nodes; and W is a set of weights (e.g., numbers), strength or intensities associated to the links (Newman, 2003; Zitnik et al., 2024). Nodes N are usually systems, relevant variables, or states of a single variable. Links usually represent interactions, couplings, relationships, probabilities, or connectivity between pairs of nodes or within nodes. Weights represent the ‘magnitude’ of the interactions, relationships, probabilities, or connectivity. Traditional complex networks represent graphs of association where nodes are static, and links are constant, and where network topology and connectivity is uniquely associated with network function. In contrast, the field of Network Physiology requires a new theoretical framework based on adaptive networks of dynamical systems (Ivanov et al., 2016). This is a new class of networks where nodes are dynamic nonlinear systems and sub-systems with transient characteristics, distinct mechanisms of regulation, and complex variability in the output signals, and where links are time-varying functional forms. Importantly, in adaptive networks of dynamical systems, global characteristics reflect the level of functionality of dynamic within-network coordination among systems, and a rich phase-space of distinct global behaviors can emerge from the same network topology due to minor temporal changes in the dynamics of individual nodes or in the functional form of network interactions (Ivanov, 2021). Novel approaches based on adaptive networks of dynamical systems are essential to address the fundamental question of how physiological states and functions at the organism level emerge out of the collective dynamics of diverse physiological systems integrated over spatio-temporal scales and levels of biological organization (Ivanov et al., 2016; Ivanov et al., 2017).

In physiological networks, links represent interactions, coordination, and sometimes the synchronization between systems and subsystems (e.g., cardio-respiratory synchronization, coupling of myoelectrical activity between two muscle groups), while network nodes represent systems and components within the physiological network (e.g., muscle groups in the intermuscular network). The weights of information transfer (i.e., strength of coupling) modulates the links within a network, with certain links acting as major mediators of interaction, while others have auxiliary roles, leading to a hierarchically structured organization of network links (Bartsch et al., 2012; Bartsch et al., 2015; Liu et al., 2015a; Lin et al., 2016; Kerkman et al., 2018; Garcia-Retortillo and Ivanov, 2022; Garcia-Retortillo et al., 2023; Rizzo et al., 2023). The weight of links informs about the flexibility and adaptivity of the studied network during exercise. When the links among the nodes stabilize in a weak or under-expressed manner (low correlation values), or conversely, overexpressed (high correlation values), the network becomes poorly adaptive. In contrast, when the network links keep flexible, rich, and moderately expressed, with adequate weight, the network is adaptive and functional.

Network-based measures, with the potential of assessing how two or more components (variables) are structurally and functionally interacting, have been used through different perspectives and frameworks in many biological and computational fields (see Rayfield et al., 2011 as an example). Some measures may assess cross-correlation, nonlinear coupling, similarity, relative phase, or other interacting properties in simpler or more complex networks (Chen et al., 2006; Xu et al., 2006; Bartsch and Ivanov, 2014; Bartsch et al., 2014; Garcia-Retortillo et al., 2026a).

Complex networks typically represent systems as statistically structured complex graphs (i.e., networks described through probabilistic or statistical features), focusing on fixed edges and global properties such as degree distribution or modularity (Albert and Barabási, 2002; Costa et al., 2007; Dorogovtsev, 2008; Gosak et al., 2018; Hawe et al., 2019). However, these approaches lack the capacity to capture time-dependent interactions and feedback mechanisms intrinsic to physiological systems. In contrast, dynamic network measures, applied from lower to higher sampling rates of the time series, treat nodes as dynamical systems and allow studying links over time, enabling a more accurate representation of the co-evolving structure and function characteristic of physiological processes (Bashan et al., 2012; Bartsch and Ivanov, 2014; Ivanov and Bartsch, 2014; Bartsch et al., 2015; Liu et al., 2015b; Ivanov et al., 2021b; Ivanov et al., 2021a).

Various measures have been developed and refined to analyze interactions in the physiology of healthy states, pathophysiology, and exercise physiology. These measures are tailored to the specific physiological questions being investigated, the characteristics of the data, and the available computational resources. Measures applied until now in exercise physiology include dimensionality reduction analyses (reducing multiple physiological variables into smaller sets of correlated variables) (Balagué et al., 2016), coherence analyses (measuring the frequency-domain correlation between two signals) (Martínez-Aguilar and Gutiérrez, 2019), Granger causality (measuring directional influences between physiological signals) (Marinazzo et al., 2008; Młyńczak and Krysztofiak, 2019), Transfer entropy (measuring dynamic causal interactions, especially in nonlinear systems) (Petelczyc et al., 2018), Mutual information (measuring the total shared information, linear and nonlinear, between two signals) (Kim et al., 2017) or O-information, synergy, and redundancy (Scagliarini et al., 2024).

Interestingly, some research has combined different measures—such as cross-correlation of instantaneous phases, coupling functions, time-delay stability, phase-amplitude coupling and phase synchronization algorithms—for capturing both simple linear relationships and more complex nonlinear dynamics, including the temporal directionality (e.g., synchronization delays) present in multivariate data (Chen et al., 2006; Bartsch et al., 2012; Bartsch et al., 2015; Stankovski et al., 2017; Rizzo et al., 2020; Ivanov et al., 2021b; Garcia-Retortillo et al., 2023; Garcia-Retortillo and Ivanov, 2025). Recently, the Amplitude-Amplitude Cross-Frequency Coupling (ACFC) method has been proposed to evaluate the oscillatory components across specific frequency bands, not only quantifying average coupling and strength of networks over extended periods of time but also the temporal variability in network interactions at short timescales (Garcia-Retortillo et al., 2026a).

To date, the development of dynamic network markers allows to mainly quantify how different key organ systems (e.g., brain, heart, skeletal muscles, etc.) coordinate and integrate as a network and how these interactions change in response to exercise, fatigue, aging, disease, etc. The following subsections introduce a set of advances and practical implications for exercise monitoring through inter-organ and inter-muscular dynamic networks:

### 2.1 Cardio-respiratory networks

Cardio-respiratory exercise testing provides valuable insights for both functional and diagnostic assessment of all types of population, from patients to athletes. The interdependent and nonlinear dynamic interaction between cardio-respiratory variables during exercise offers a great opportunity to evaluate health and fitness states (Abreu et al., 2023). Commonly, such cardio-respiratory variables are assessed by ECG and gas exchange analyzer, respectively, although non-contact techniques like photoplethysmography imaging and respiratory data through optical flow method have also been proposed (Borik et al., 2023).

Cardio-respiratory coupling was the first studied dynamic organ interaction during exercise (Schäfer et al., 1998; Schäfer et al., 1999). Such interaction, affected by different neural and mechanics factors like respiratory sinus arrhythmia, cardioventilatory coupling and respiratory stroke volume synchronization (Dick et al., 2014; Elstad et al., 2018), has been quantified through several measures. From univariate linear measures based on frequency domain of heart period driven by respiration, bivariate analyses measuring the coupling between HR and respiratory phases, to multivariate analyses across different cardio-respiratory variables (Abreu et al., 2023).

The application of coherence linear analysis calculated on bivariate time series of heart and respiratory rhythms has permitted to reveal, for instance, a higher cardio-respiratory coupling strength at rest in athletes than non-athletes associated with higher exercise capacity (VO_2peak_) (Abreu et al., 2024), as well as in high-level runners compared to middle-level runners when exposed to hypoxia (Uryumtsev et al., 2020).

Techniques like the classic respiratory sinus arrhythmia, or other measures of nonlinear interaction such as phase-synchronization analysis or time-delay stability analysis, seem to sensitively inform together about the loss of link strength and time synchronization of cardio-respiratory coupling in different physiological states (Bartsch et al., 2012; Bartsch et al., 2014; Bartsch and Ivanov, 2014). These studies suggested that cardiac-respiratory communication occurs in alternating “on” and “off” periods and that different forms of coupling can coexist, each reflecting distinct aspects of physiological regulation. Some of these techniques have been useful in assessing, for example, how cardio-respiratory coordination might increase after a period of high-intensity training in endurance athletes (De Meersman, 1992; Al-Ani et al., 1996) or after training aimed at promoting rhythmic and controlled breathing in exercise participants (Perry et al., 2020).

Other nonlinear measures such as dynamical Bayesian inference analysis of bivariate phase interactions or multimodal coupling analysis have also been applied to ECG and respiratory signals at rest to detect decrease of cardio-respiratory coupling in unhealthy populations (Lin C. et al., 2020; Sorelli et al., 2022). Transfer entropy allowed also to detect changes of connectivity between different cardiorespiratory variables during exercise and hypoxia conditions in exercise participants, showing how heart rate and oxygen saturation were important hubs in front such respective stressors (Morandotti et al., 2025).

Finally, multivariate analyses were applied during cardio-respiratory testing to assess cardio-respiratory coordination by reducing the dimensionality of time series through principal component analysis (Balagué et al., 2016). This technique, evaluating the mutual influences between cardiovascular and respiratory oscillations across various variables (e.g., expired O2 and CO2 fractions, ventilation, systolic and diastolic blood pressure, and HR), has been effectively used in studies of fatigue (Garcia-Retortillo et al., 2017), training effects (Garcia-Retortillo et al., 2019), nutritional interventions (Esquius et al., 2019), pathological conditions (Oviedo et al., 2021), performance levels (Papadakis et al., 2022), and fitness levels (Abenza et al., 2025b). However, this technique does not account for temporal directionality and nonlinear dynamic outcomes (Schulz et al., 2013; Abreu et al., 2023).

Cardio-respiratory networks extracted from cardio-respiratory testing have been approached with certain limitations. Promising network-based measures like time-delay stability or Bayesian inference analysis need to be further investigated in cardio-respiratory exercise testing. Understanding the transient and nonlinear nature of cardio-respiratory coupling may have significant practical implications. Insights into the stability and synchronization patterns of the cardio-respiratory systems during exercise could contribute to the early detection and prevention of exercise-induced disorders such as overtraining. Thus, future research exploring the coupling between the cardiovascular and respiratory systems is warranted to improve its applicability in health and performance fields.

### 2.2 Inter-muscular networks

Inter-muscular coordination, referring to the synchronization between different muscles that enables smooth and effective movement patterns to achieve task goals, has been a major focus of research (Haken et al., 1985; Kelso and Scholz, 1985; Prilutsky, 2000; Ting et al., 2015; Garcia-Retortillo and Ivanov, 2022).

Such coordination has been mainly approached through two models: i) muscle synergies, and ii) intermuscular coherence, both establishing a solid background on the mechanisms underpinning motor control. Muscle synergies models are based on dimensional reduction or matrix factorization measures informing about the shared information between EMG signals, and thereby, how muscles coordinate at specific activation patterns (timing or amplitude) (e.g., d’Avella and Bizzi, 2005; Ting et al., 2015; Kerkman et al., 2020; Kerkman et al., 2022; Cheung and Seki, 2021). While intermuscular coherence generally looks for the coupling between determined frequency bands (iso-frequency) of EMG, associating frequency domains to determinate neural inputs of the central nervous system (e.g., Boonstra et al., 2008; Kattla and Lowery, 2010; Kerkman et al., 2018; Weersink et al., 2021; Maillet et al., 2022; Rossato et al., 2022).

However, muscle synergy models inherently limit the dimensionality of neural control to be no greater than the number of recorded muscles and assume that all motor neurons within a motor pool (i.e., the collection of motor neurons innervating a single muscle) receive identical inputs (Hug et al., 2023). This implies that the smallest analytical unit in the synergy-based model is the entire muscle (d’Avella and Bizzi, 2005; Ting et al., 2015). Alternatively, the central nervous system controls specific clusters of motor neurons across different motor pools, both within and between muscles, rather than regulating the overall muscle dynamics (Hug et al., 2021; Vecchio et al., 2023).

Synergy and coherence-based metrics mostly capture the linear interactions between same frequency bands (iso-frequency coupling between muscles) and cannot measure nonlinear coupling across different frequency bands (Yang et al., 2018; Garcia-Retortillo and Ivanov, 2022; Garcia-Retortillo et al., 2023). Consequently, these approaches may overlook valuable information regarding the interaction between distinct EMG frequency bands. Cross-frequency coupling between various frequency bands and the continuous flow of information within a network of frequencies may be essential for integrating both local and global processes, promoting adaptability to inputs, and supporting a range of dynamic behaviors and physiological states (Jirsa and Müller, 2013; Liu et al., 2015a; Lin et al., 2016; Lin et al., 2020a; Rizzo et al., 2020; Chen et al., 2022). Furthermore, coherence-based metrics may often represent static networks without using time series, and therefore, ignore dynamic networks assessment. Temporal directionality is also not generally included in these metrics.

To enhance our understanding of muscle coordination and motor control, and to complement traditional approaches like synergy and coherence analysis, ACFC has been used to explore intermuscular cross-frequency interactions among different muscles while accounting for their nonlinear dynamics and directionality. This measure has proven effective in analyzing how distinct frequency bands within muscle dynamics (i.e., representing distinct motor recruitment patterns and other neuromuscular characteristics) synchronize and form a network across muscles during physical activity, as well as in tracking how these intermuscular interactions adapt with fatigue in a healthy population (Garcia-Retortillo et al., 2020; Garcia-Retortillo et al., 2023; Garcia-Retortillo and Ivanov, 2022). Studies have further investigated its application in older adults (Garcia-Retortillo et al., 2025a; Garcia-Retortillo et al., 2025b) and in examining sex differences in young exercise participants (Abenza et al., 2025a).

From a practical perspective, the mentioned approaches can be used to create network-based markers to assess and quantify inter- and intra-muscular interactions in various exercises and different cohorts and individuals. Identifying the global inter-muscular network during exercise and their reorganization in response to perturbations like fatigue might be crucial for addressing some limitations of traditional physiological parameters used to assess health and fitness (Balagué et al., 2024). For instance, to help in the prevention and diagnosis of neurodegenerative disorders and falls in older adults, muscle injuries or even as a training workload marker. Future studies in inter-muscle networks might combine EMG and near-infrared spectroscopy (NIRS) to study the interaction of electrical activity and oxygenation capacity within and between working muscles.

### 2.3 Cardio-muscular networks

Earlier studies established the fractal and stochastic organization of locomotor dynamics (Hausdorff et al., 2001; Ashkenazy et al., 2002; Hu et al., 2004), while subsequent work examined how cardiovascular and locomotor functions interact during exercise (Kirby et al., 1989; Niizeki et al., 1993; Nomura et al., 2003; Constantini et al., 2018). This phenomenon suggests that HR and step rate (the number of steps taken per unit of time) naturally synchronize during rhythmic movement, maintaining a consistent ratio (Phillips and Jin, 2013). Aligning the timing of cardiac and locomotor phases optimally may improve hemodynamic efficiency and cardiovascular function during rhythmic exercise, potentially benefiting heart health (Walløe and Wesche, 1988; Kirby et al., 1989; Wakeham et al., 2023). For example, coordinating foot strikes with the diastolic phase of the cardiac cycle, rather than the systolic phase, can lead to a significant reduction in HR during exercise and promote positive metabolic effects (Constantini et al., 2018; Wakeham et al., 2023). Regarding the hemodynamic, it has been also demonstrated how changes in HR are related to the desaturation and resaturation of muscle oxygenation during exercise (Manchado-Gobatto et al., 2022; Vasquez-Bonilla et al., 2024).

While these studies have provided very relevant insights into the relationship between HR with step rate or muscle oxygenation, they do not investigate the dynamic interaction between cardiac and muscle activities. They focus on discrete “snapshots” to evaluate physiological outcomes through phase timing between HR and step rate or NIRS-derived variables, without assessing how direct cardio-muscular coupling evolves during exercise. As widely known, cardiac activity is closely interrelated to muscle activity to guarantee energetic pathways, and this dynamic interaction might importantly inform about the biological reorganization that permit moving.

Some research has been done comparing the ECG-EMG coherence during exercise, demonstrating the reduction of cardio-muscular coupling with fatigue in inactive population (Pichardo-Rivas and Gutiérrez, 2021). Moreover, Petelczyc et al. (2018) demonstrated in trained athletes through Bivariate transfer entropy between R-R intervals and muscle deoxygenation that: at lower exercise intensities the coupling directionality between both variables is dominated by muscle deoxygenation (important role of metaboreceptors), while at higher intensities the direction was reversed (important role of baroreceptors and mechanoreceptors).

On the other hand, recently cardio-muscular coordination has been introduced as a novel analytical approach beyond cardio-muscular coherence, analyzing the degree of cross-correlation between time series of instantaneous HR and EMG spectral power frequency bands (ACFC) (Garcia-Retortillo and Ivanov, 2025; Langan, 2025). Garcia-Retortillo and Ivanov (2025) revealed that, in young exercise participants, the heart operates in synchrony with muscles to maintain homeostasis, facilitate movement, and adapt to exercise and fatigue demands, although the exact mechanisms driving this cardio-muscular coupling remain unclear. Findings indicate a notable decrease in cardio-muscular coupling with fatigue, with complex transitions from synchronous to asynchronous behaviour.

From a practical point of view, the quantification of dynamic cardio-muscular interactions has the potential to assess global health, levels of fatigue, fitness status or the effectiveness of cardiovascular and muscle injury rehabilitation programs. Further research may include multivariate analyses uncovering the linear and nonlinear coupling, as well as temporal directionality, among ECG, EMG and NIRS-derived variables.

### 2.4 Cortico-muscular networks

Cortico-muscular coordination—the bidirectional communication between the central nervous system and peripheral muscles—has long been a central focus in neuromuscular physiology and motor control research (Stein, 1974; Kelso and Stelmach, 1976; Kelso, 1977; Bernstein, 2014). Aside from the interesting molecular crosstalk between both organs, such as how some myokines might favor brain plasticity (Isaac et al., 2021); this interaction has been traditionally investigated regarding their dynamics using coherence analyses between EEG and EMG signals (Mima and Hallett, 1999). These studies have primarily focused on linear, frequency-specific coupling, most notably in the β (beta) band (13–30 Hz), which is associated with sustained motor output and sensorimotor integration (Conway et al., 1995; Kilner et al., 2000). Such cortico-muscular coherence (CMC) reflects a functional link between motor cortical areas and spinal motor neurons, providing insight into how the brain modulates muscle activity during tasks like isometric contraction, walking, or skilled voluntary movement (Gwin and Ferris, 2012; Roeder et al., 2020; da Silva Costa et al., 2024). However, brain and muscle synchronization involve short bursts of frequency-specific connectivity (i.e., motor beta activity is transient) even when task demands are sustained (Echeverria-Altuna et al., 2022).

Research examining CMC during exercise has expanded the understanding of this brain-muscle connectivity across various frequency bands (δ, θ, α, σ, and β), yielding important insights for both health and athletic performance (Boonstra et al., 2009; Mehrkanoon et al., 2014; Liu et al., 2019; Santos et al., 2024). For example, it has been demonstrated increased CMC between the motor cortex and both agonist and antagonist muscles during exercise in different trained populations (Dal Maso et al., 2017; Dal Maso et al., 2018). Fatigue-induced reductions in CMC suggest a possible disintegration of communication between the sensorimotor cortex and muscles in healthy populations (Martínez-Aguilar and Gutiérrez, 2019), while lower CMC values in older or unhealthy populations indicate impaired neuromuscular coordination (Cremoux et al., 2017; Roeder et al., 2020). CMC also reflected inter-individual differences in the state of cortico-muscular network when learning motor tasks (Mendez-Balbuena et al., 2012).

Different techniques have been used to approach brain-muscle connectivity, from bivariate analysis (e.g., coherence analysis, Granger causality) examining the interaction between one EEG channel and one EMG channel (Witham et al., 2010); to multivariate analysis (e.g., Dynamic causal modelling, Non-negative matrix factorization, Partial directed coherence analysis) simultaneously examining multiple EEG and EMG channels (Artoni et al., 2023; Roeder et al., 2024; Wang et al., 2024). Using multivariate analysis it has been shown how CMC was higher in eccentric and concentric compared to isometric movement periods during squats (Kenville et al., 2020). Further, nonlinear measures like Transfer entropy (Vicente et al., 2011; Ke and Luo, 2024) or Mutual information (Kim et al., 2017) have been used to infer directional and nonlinear features of cortico-muscular interactions. For example, using Mutual information, higher cortico-muscular interaction and shorter time delay was evidenced when performed active exercise with movement intention than performed passive exercise without intention (Kim et al., 2017).

Adding to this emerging techniques, recent findings have highlighted the fundamental principles of neuro-autonomic regulation of muscle activation across sleep-wake states, which are very relevant for training recovery. Using time-delay stability and other temporal-directional analyses, studies have shown that cortico-muscular communication reflects rhythmic, state-dependent patterns of synchronization between brain rhythms and peripheral muscle activity (Liu et al., 2015a; Lin A. et al., 2020; Rizzo et al., 2020; Rizzo et al., 2022; Rizzo et al., 2023; Ivanov and Bartsch, 2022; Ivanov and Bartsch, 2025). These findings suggest that the organization of brain-muscle networks encodes physiological state and may deteriorate in pathological conditions. Such dynamic coupling patterns across multiple EEG bands and EMG frequencies underscore the need for approaches that can handle nonlinearity, directionality, and multiscale temporal complexity during exercise.

Because of the brain’s complex hierarchical and modular architecture, with nodes connecting within and between modules in a collaborative manner to simultaneously integrate multisensory information, it will be very valuable in the future to acquire a more integrated approach (Guimerà and Amaral, 2005; Sporns et al., 2007; Zamora-López et al., 2010; Zamora-López et al., 2011). Not only within and between both hemispheres (Tecchio et al., 2006; Zamora-López et al., 2011), but especially accounting for the other non-cortical regions (e.g., cerebellum, hypothalamus, etc.) with over 80% of brain neurons (Azevedo et al., 2009) and important roles in motor coordination and adaptive control (Thach, 1998; Bostan and Strick, 2018). In particular, considering how global brain connectivity is subcortically dominated linking cortex and cerebellum networks, this region involves an important role for the communication and integration between different brain areas (Schulte et al., 2025). Future research should therefore prioritize inclusion of these regions, alongside multivariate and temporally-sensitive measures, to elucidate how complex brain-muscle networks behave during exercise and it is affected by fatigue, aging, neurodegeneration, sex, among others. Additionally, taking into account also the neural cross-frequency coupling functions extracted from multivariate networks (Stankovski et al., 2015).

Finally, future research could extend beyond EEG and EMG signals to include brain and muscle oxygenation dynamics measured through fNIRS and NIRS (Chen et al., 2024). Notably, the patterns of EMG and fNIRS signals during exercise appear to be positively correlated, with stronger correlations observed in individuals with healthier lifestyles (Daniel et al., 2023).

### 2.5 Cortico-cardiac networks

The interaction between brain and heart is key for multiple physiological functions such as autonomic regulation, baroreceptor reflex, neuroimmune-heart axis, muscle contraction or even emotional states (Yu X. et al., 2018; Schnabel et al., 2021; Liu et al., 2022; Catrambone and Valenza, 2023; Valenza et al., 2025). The complex brain-heart axis is communicated by neural, mechanical and biomechanical pathways involving, for example, how blood pressure changes or protein mediators of acute and chronic inflammation modulate the functioning of both organs (Fan et al., 2024; Valenza et al., 2025). They also seem to be associated by their structure and genetic colocalization, suggesting that adverse heart metrics may have implications for brain diseases (Zhao et al., 2023). Despite brain-heart crosstalk has been primarily focused on static snapshots, research focused on their dynamic interactions through the lens of network-based frameworks are especially increasing around diseases and psychobiological states but is still scarcely applied to exercise.

Heartbeat-evoked potential has been used as a common technique to study how the EEG responses to the heartbeat (time-locked to R-peaks) (Montoya et al., 1993). This method has shown, for example, how meditation induce the reorganization of cardiac influenced neuronal activity and the underlying large-scale brain networks such as cortical plasticity (Jiang et al., 2020). Nonetheless, this method only captures how the brain responds to cardiac output but not the other way around. Correlation analyses have also been used to show positive correlation between heart rate variability at high-frequency and EEG frontal midline *θ* band power after meditation (Tang et al., 2009), or between the heart rate with the brain hemodynamic (fNIRS) when focusing on heartbeat counting (Candia-Rivera et al., 2022).

More advanced techniques like Maximal information coefficient, Time-delay stability, entropy measures or Granger causality have been effectively used to assess linear and nonlinear cortico-cardiac couplings, in some cases studying the role of different frequency bands, to demonstrate the arousal, emotional and sleep phases dependency (Faes et al., 2014; Faes et al., 2015b; Valenza et al., 2016; Abdalbari et al., 2022; Cai et al., 2024). In this line, Catrambone et al. (2019) shown how cold stress increased the magnitude of the brain-heart coupling (EEG bands and heart rate variability), firstly from brain to heart and after some time delay (about 60 s) changing from heart to brain.

In the field of exercise few research on brain-heart interaction have been performed. Martínez-Aguilar and Gutiérrez (2019) showed how cortico-cardiac maximum coherence, in the β band, changed in fatigued conditions and suggested, similarly than cortico-muscular interaction, a loss of connectivity between both organs when effort is accumulated. Further, Pichardo-Rivas and Gutiérrez (2021) could explore through Partial directed coherence how sedentary population, compared to active population, had less connectivity between the frontal brain cortex, the heart and the muscle in the process of fatigability. This demonstrates how active people in front of fatigability comes with an increase in cardiac and muscular self-regulatory processes.

In view of the above, further research on brain-heart dynamic interaction is warranted to give valuable applications in exercise and sport monitoring, such as enhancing motor learning processes or detecting health disorders associated with network connectivity failures (e.g., sudden death; Lerma and Glass, 2016). In addition, as mentioned previously, deeper analyses of the nonlinear dynamics and temporal directionality governing multilevel network functions are needed, considering the complex hierarchical and modular architecture of the brain.

## 3 Discussion

### 3.1 Scope and current evidences

Conceiving athletes as complex adaptive systems suggests that monitoring should not rely exclusively on isolated and static variables, but also consider the dynamic coordination and integration of multilevel psychobiological processes.

Network Physiology provides a conceptual and analytical framework to assess interactions among physiological systems during exercise. Studies applying this framework across organ systems (e.g., heart, lungs, muscles, brain), have shown that network-based measures can capture dynamic coordination patterns among physiological signals and are sensitive to relevant phenomena such as fatigue, and detect aging effects, sex differences, and pathological conditions. These findings support the relevance of studying exercise responses as emerging properties of interacting physiological systems rather than as independent processes (Bashan et al., 2012; Bartsch et al., 2015; Ivanov et al., 2016; Balagué et al., 2020; Balagué et al., 2022a).

In particular, the analysis of temporal variability and coupling among physiological signals has revealed important information about how physiological systems coordinate during exercise. Previous research has widely shown that the variability of isolated physiological variables can reflect adaptive capacity and system flexibility in response to exercise and training (Nunes Amaral et al., 2001; Karasik et al., 2002; Lin et al., 2015; Biltz and Lundstrom, 2023). More recent work suggests that examining the temporal dynamics of interactions among physiological nodes may provide additional information about how physiological networks reorganize during exercise (Abenza et al., 2025a; Garcia-Retortillo and Ivanov, 2025). For instance, Abenza et al. (2025a) have shown that temporal variability of intermuscular networks was key to capturing sex differences during exercise. This dynamic network approach seems promising for solving a crucial and still unsolved question about distinguishing responders and non-responders to exercise, and providing relevant information for personalizing adequately exercise interventions oriented to health and performance.

While existing studies demonstrate the feasibility of analyzing physiological networks during exercise, the translation of these approaches into practical monitoring tools for health or performance purposes remains limited.

### 3.2 Limitations and future challenges

Current applications of Network Physiology to exercise primarily characterize dynamic inter-organ coordination and, therefore, capture selected levels of the multiscale physiological organization underlying exercise responses. As depicted in Figure 1, organ interactions may reorganize dynamically during increasing exercise intensity, potentially reflecting progressive changes in coupling among physiological systems.

**FIGURE 1:**
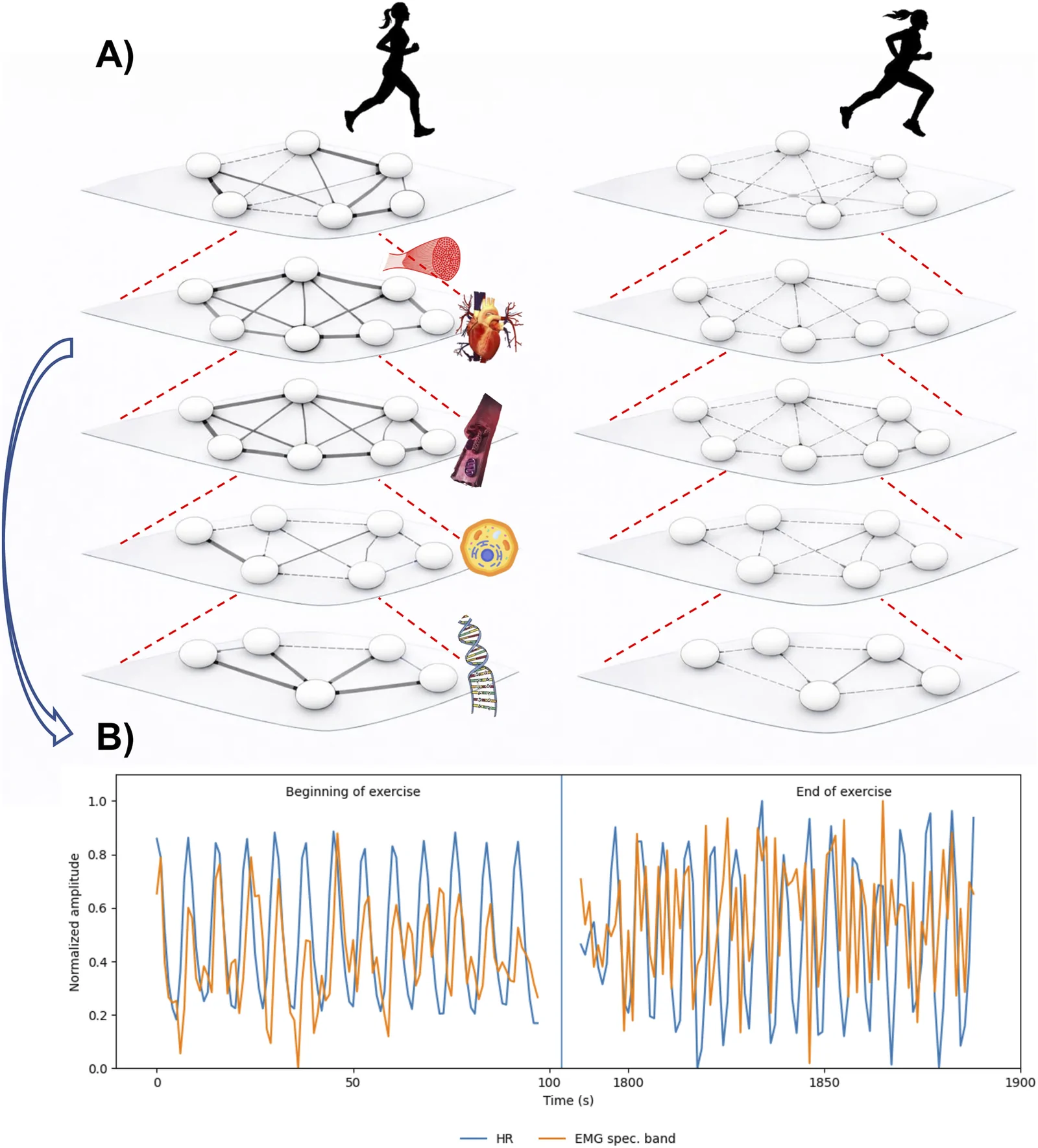
Conceptual model illustrating how multilevel physiological networks may hypothetically reorganize during an incremental exercise. Up **(A)** The figure depicts a progressive loss of connectivity across horizontal and vertical levels (organs, tissues, cells, genes) as exercise intensity increases. Down **(B)** normalized time series at 1Hz from heart rate (HR) and electromyography of a specific frequency band (EMG spec. band) from vastus lateralis displaying relatively coupled dynamics at the beginning of the exercise (left side) and uncoupled dynamics at the end (right side) (inspired in Garcia-Retortillo and Ivanov, 2025).

A key limitation concerns the simultaneous acquisition of high-quality physiological signals across multiple biological levels. Building a comprehensive dynamic physiological map would require synchronized, high-resolution recordings of multiple variables operating across different timescales. As illustrated conceptually in Figure 1, a future possibility within the Network Physiology framework is the study of dynamic vertical interactions across biological levels (e.g., genes, cells, tissues, organs) and their coordination during exercise (Balagué et al., 2022b). However, such vertical integration remains largely unexplored due to current technological limitations in recording physiological processes across these levels with sufficient temporal resolution.

In particular, omics techniques (genomics, proteomics, metabolomics, etc.) provide valuable integrated molecular data (e.g., myokines, inflammatory proteins) to better understand athletes’ states such as hypertrophy, training responsiveness, or injuries (Hoffman, 2017; Rodas et al., 2020; Bassini et al., 2022; Sellami et al., 2022). Despite these advances, most current models rely on complex network representations based on graph theory, which offer only static snapshots of molecular associations (Balagué et al., 2022a; Balagué et al., 2022b). Current technologies do not yet allow the acquisition of high-frequency time series data at such microscopic scales. Consequently, a future challenge for omics is to solve such temporal resolution and develop dynamic network markers (Nilsson et al., 2006; Hu et al., 2016; Berry et al., 2022; Amar et al., 2024).

The analysis of physiological networks also presents methodological challenges. Network-based approaches may include analytical techniques capable of capturing nonlinear dynamics, time-varying interactions, and multivariate dependencies among physiological signals. However, these analyses are constrained not only by limited sampling and temporal resolution but also by their susceptibility to noise, artifacts, and variability in signal quality; factors that can substantially bias inferred network structure and dynamics, especially in non-stationary physiological networks where intrinsic fluctuations and measurement noise interact with time-varying coupling mechanisms (Xifra-Porxas et al., 2021; Laumann et al., 2024; Scarciglia et al., 2025). Moreover, methodological standardization and reproducibility remain important challenges, as different network inference techniques may lead to different representations of physiological interactions (Bashan et al., 2012; Ivanov and Bartsch, 2014; Bartsch et al., 2015).

A shift toward a dynamic network approach may provide a more fundamental, hierarchically organized understanding of physiological adaptation to exercise. Within this perspective, interactions among physiological systems can be explored across multiple biological levels and timescales, potentially revealing common organizational principles underlying exercise responses. For example, the presence of statistical dilation symmetry, whereby system dynamics exhibit similar patterns across different temporal scales (Falconer, 1994; Hristovski, 1996), may help link markers of critical behaviour observed during both acute (Hristovski and Balagué, 2010) and chronic fatigue states (Tian et al., 2013). Advancing this line of research will require identifying network interactions that are sensitive to specific physiological states and transitions.

This effort will likely involve systematic investigation and modelling of nested network interactions, including both horizontal interactions among organ systems (e.g., lungs–muscles as recently studied by Garcia-Retortillo et al. (2026b), eyes–muscles, gastrointestinal–brain interactions, etc.) and vertical interactions across biological levels. Although brain networks were not specifically addressed in the present work due to their extensive prior investigation (Rossini et al., 2019; Büchel et al., 2021; Li et al., 2022), integrating neural dynamics with whole-body physiological networks remains an important direction for future research. Accordingly, network-based measures could support more personalized and adaptive decision-making while fostering collaboration among interdisciplinary teams that still often work from fragmented evaluations.

Further work is also needed to explore how key complex systems properties—such as variability, hysteresis, degeneracy or pleiotropy—manifest within physiological networks during exercise and recovery. For example, while investigating the hysteresis of networks would offer valuable insights into both the recovery efficiency and inertia of physiological states (Montull et al., 2020; Montull et al., 2023; Leacy et al., 2021); the assessment of network degeneracy and pleiotropy would help to detect key physiological compensating roles among physiological systems (Latash, 2024).

Interpretative and validation challenges further limit the practical application of network-based monitoring. Further research is warranted to identify the most sensitive network markers to inform about relevant exercise-related phenomena such as fatigue, injury susceptibility, overtraining, or maladaptive responses, while avoiding excessive or redundant monitoring of variables.

Artificial intelligence and machine learning approaches may support the development of network-based measures by identifying patterns within large multivariate data. In particular, leveraging intra-individual network variability to identify stability-instability network patterns and enhance the probability to detect organismic reconfigurations. However, the development of such models will require extensive physiological validation to ensure that detected patterns correspond to meaningful physiological processes rather than statistical artefacts (Yu K. H. et al., 2018). Consequently, AI-based approaches should currently be considered exploratory tools rather than ready-to-implement monitoring systems.

Finally, a key challenge for exercise monitoring is to complement such conceptual, methodological and technological development moving towards more integrative and dynamic tools based on subjective data extracted from questionnaires and interactive interviews; an immediately available tool with an enormous but often underestimated potential of compressing in real time multiple levels of organism-environment information (Montull et al., 2022).

## 4 Conclusion

Network Physiology provides the conceptual and analytical framework to investigate how physiological systems dynamically interact and organize as a network, and how physiological networks adapt and evolve with transitions across states and change in conditions to facilitate functions at the organism level. Applied to exercise, this framework extends monitoring beyond isolated variables toward time-varying and nonlinear interactions among multiple diverse physiological systems. Future work should focus on methodological standardization, multilevel data integration and physiological validation of network markers for health, disease and performance monitoring.

## Data Availability

The original contributions presented in the study are included in the article/supplementary material, further inquiries can be directed to the corresponding authors.

## Footnotes

1 Synergies are sets of mutually compensating system components which covariate in order to satisfy some functional goal constraints. Components may be represented as network nodes and their covariation as links of the network. The covariation necessarily brings about a dimension reduction of the degrees of freedom of the system (Scholz and Schöner, 1999). When defining synergies, it is important to emphasize that the covariation is always measured and formally connected to the goal performance variable. Synergies are key physiological mechanisms of enabling functionality of biological systems.
